# Synthesis, characterization, and preliminary insights of ZnFe_2_O_4_ nanoparticles into potential applications, with a focus on gas sensing

**DOI:** 10.1038/s41598-023-46960-w

**Published:** 2023-11-11

**Authors:** Zeyad M. Abdulhamid, Aasif A. Dabbawala, Thomas Delclos, Rainer Straubinger, Magnus Rueping, Kyriaki Polychronopoulou, Dalaver H. Anjum

**Affiliations:** 1 Department of Physics, Center for Catalysis and Separations (CeCaS Center), Khalifa University of Science and Technology, Abu Dhabi, 127788 United Arab Emirates; 2 Department of Mechanical Engineering, Center for Catalysis and Separations (CeCaS Center), Khalifa University of Science and Technology, Abu Dhabi, 127788 United Arab Emirates; 3 Manager, Materials, and Surface Core Labs, Khalifa University of Science and Technology, Abu Dhabi, 127788 United Arab Emirates; 4 Core Technology Platforms, New York University Abu Dhabi, Abu Dhabi, 129188 United Arab Emirates; 5 KAUST Catalysis Center (KCC), King Abdullah University of Science and Technology, 23955-6900 Thuwa, Saudi Arabia

**Keywords:** Condensed-matter physics, Nanoscale materials

## Abstract

This work presents a hydrothermal-based facile method for synthesizing ZnFe_2_O_4,_ whose size can be controlled with the concentration of sodium acetate used as a fuel and its physical changes at nanoscales when exposed to two different gases. The structural, morphological, compositional, and electronic properties of the synthesized samples are also presented in this paper. The crystal structure of the synthesized samples was determined using an X-ray Diffractometer (XRD). The results revealed fluctuations in the size, lattice parameter, and strain in the nanoparticles with increasing the concentration of sodium acetate. Field-Emission Scanning Electron Microscopy (FESEM) was used to determine synthesized materials’ morphology and particle size. It revealed that the particles possessed approximately spherical morphology whose size decreased significantly with the increasing amount of sodium acetate. Transmission Electron Microscopy (TEM) was utilized to determine the structure, morphology, and elemental distributions in particles at the nanoscale, and it confirmed the findings of XRD and FESEM analyses. The high-resolution TEM (HRTEM) imaging analysis of the nanoparticles in our studied samples revealed that the particles predominantly possessed (001) type facets. X-ray photoelectron spectroscopy (XPS) and core-loss electron energy loss spectroscopy (EELS) showed an increasing fraction of Fe^2+^ with the decreasing size of the particles in samples. The Brunauer, Emmett, and Tellers (BET) analysis of samples revealed a higher surface area as the particle size decreases. In addition, the determined surface area and pore size values are compared with the literature, and it was found that the synthesized materials are promising for gas-sensing applications. The ab initio calculations of the Density of States (DOS) and Band structure of (001) surface terminating ZnFe_2_O_4_ were carried out using Quantum Espresso software to determine the bandgap of the synthesized samples. They were compared to their corresponding experimentally determined bandgap values and showed close agreement. Finally, in-situ TEM measurement was carried out on one of the four studied samples with robust properties using Ar and CO_2_ as reference and target gases, respectively. It is concluded from the presented study that the size reduction of the ZnFe_2_O_4_ nanoparticles (NPs) tunes the bandgap and provides more active sites due to a higher concentration of oxygen vacancies. The in-situ TEM showed us a nanoscale observation of the change in one of the crystal structure parameters. The d spacing of ZnFe_2_O_4_ NPs showed a noticeable fluctuation, reaching more than 5% upon exposure to CO_2_ and Ar gases.

## Introduction

Ferrite is a general term used on any ferrimagnetic ceramic material. According to the crystal structure, there are three types of ferrites: Garnet, Hexagonal, and Spinel. Our work will focus on the spinel one, which has a cubic system with a chemical formula MFe_2_O_4_ where M is any divalent metal ion, and Fe is a trivalent metal ion. It is usually the primary reason for the magnetic properties of this material. Spinel ferrites exhibit great interest from researchers due to their importance in gas sensing, transformer cores, batteries, power supplies, and biomedical applications^1^. Their ability to be doped with different metal ions, chemical stability, and band gap tunability make them very promising for gas sensing^2^. Spinel ferrites with zinc Zn^2+^ as a divalent metal ion are called zinc spinel ferrites. Bulk zinc spinel ferrite belongs to cubic spinel structure under $Fd\overline{3}m$ space group and lattice parameter a = 8.35 Å. It has a typical spinel structure where Zn^2+^ ions occupy the A-sites and Fe^3+^ ions occupy the B-sites^3^. ZnFe_2_O_4_ is an n-type semiconductor with high electronic conductivity (~ 10^5^ Ω^−1^ cm^−1^)^4^ in its bulk scale. It stands out from the other ferrites due to its low cost, good chemical stability, low eddy current loss, environmental-friendliness, non-toxicity, and high theoretical capacity (~ 1000 mA h g^−1^)^5–8^. Due to all these desirable properties, ZnFe_2_O_4_ could be introduced in various technological applications, as mentioned above. For instance, the capability of ZnFe_2_O_4_ NPs for detecting multiple gases such as C_2_H_6_OH, H_2_S, and NO_2_ were recorded and showed an enhancement result^9–11^. It was found that the gas response could reach its highest values at high operation temperatures (˃200 °C). Also, changing base precursors could cause a particle size reduction varied from 23.9 nm for NH_4_OH to 21.6 nm and 16.2 nm for LiOH and KOH, respectively^9^. This reduction in particle size could increase the surface area and hence enhance the gas sensitivity. On the other hand, extensive exploration into the inherent connection between morphology and size with gas sensing properties has underscored the critical necessity for adaptable synthesis techniques. These methods aim to offer control over the size and morphology of ZnFe_2_O_4_ while incorporating specific functionalities. Consequently, various methodologies have been applied to synthesize ZnFe_2_O_4_ nanostructures featuring varied morphologies, such as nanoparticles^12^, nanorods^13^, nanoflowers^14^, and nanospheres^15^. These wide morphologies that could be derived from ZnFe_2_O_4_ are due to its feasibility to be synthesized with various synthesis methods, including sol–gel, microwave, hydrothermal, and coprecipitation^16^. Numerous studies in gas sensors have indicated that sensing materials characterized by porous structures can significantly enhance their gas-sensing capabilities. This improvement is attributed to the larger surface area, reduced density, and improved surface permeability inherent in these materials^17^. Besides, changing synthesis conditions such as annealing temperature and fuel or base precursor could alter the particle size and shape effectively. Based on all the above, this work aimed to provide three key points: First, synthesize spherical-like ZnFe_2_O_4_ NPs with different nanoscale sizes by changing the base precursor or fuel concentration using the hydrothermal method. The fuel that could meet our requirements without causing lattice distortion or foreign phase is sodium acetate. At the same time, the reason for choosing the hydrothermal method is its versatility, which can produce high-purity and homogeneous NPs. Also, it is a cost-effective and non-toxic technique. Second, performing a detailed structural, morphological, and physical analysis as well as chemical states on the macroscale using XPS and nanoscale using EELS. Third, testing one of the four samples with robust properties via in-situ TEM under CO_2_ and Ar atmospheres. This test was done under 300 °C operating temperature, where our studied material became chemically active as previously reported. This technique enabled us to ensure that ZnFe_2_O_4_ NPs with critical size would be beneficial for gas sensing applications. The gas sensing in this technique is done by monitoring or observing the change in one of the nanoscale crystal structure parameters upon exposure to Ar and CO_2_ at different time durations. However, most gas sensing techniques are based on changing a macroscale physical property, such as resistance after exposing the gas, without observing this change in the nanoscale. For instance, Kuebel et al. used the TEM beam to reduce the SiO inside the TEM and measure the resistance. Due to a lack of an in situ gas cell with an electrical connection, they removed the sample from the TEM to expose it to oxygen and change the structure to SiO^18^. Moreover, Staerz et al. illustrated how the combination of in situ microscopy and operando spectroscopy can be utilized to clarify the sensing mechanism of metal oxides^19^. Carbon dioxide, a potent greenhouse gas, is experiencing persistent escalation within the atmosphere, consequently contributing to the predicament of global warming. A captivating and potential remedy to mitigate CO_2_ emissions involves its conversion into value-added commodities, including fuels and everyday chemical products^20^. Among the diverse methodologies, solar-driven thermochemical CO_2_ reduction stands out as a practical approach, wherein the formidable C=O bond is broken, leading to carbon monoxide (CO) formation^21^. So, CO_2_ is a probe gas of high interest, where Ar was used as a reference atmosphere.

## Experimental section

### Material synthesis

**Figure 1:**
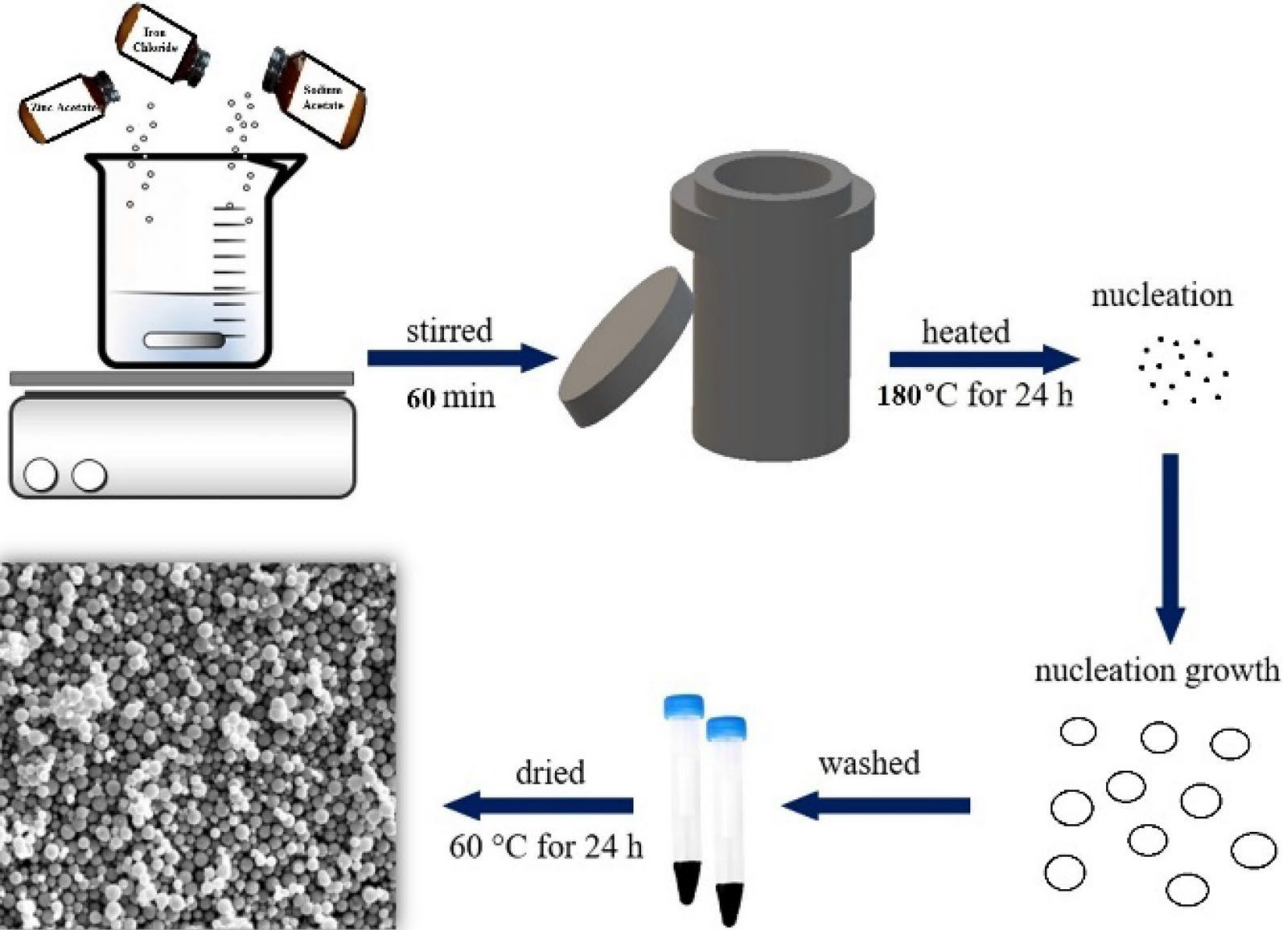
Schematic diagram for the hydrothermal synthesis of ZnFe_2_O_4_ NPs.

ZnFe_2_O_4_ was synthesized using the hydrothermal method. A schematic diagram for synthesizing ZnFe_2_O_4_ NPs is shown in Fig. 1. Sodium acetate was used to control the particle size and added to each synthesis in different molar amounts. 2 mmol zinc acetate [Zn(CH_3_COO)_2_·2H_2_O with M.W: 219.51 gm mol^−1^ purity: 99.5%], and 4 mmol of iron chloride [FeCl_3_·6H_2_O with M.W: 270.33 gm mol^−1^ purity: 99%] were used as starting materials. Then, different molar amounts of sodium acetate [CH_3_COONa·3H_2_O with M.W: 136.08 gm mol^−1^ purity: 99.5%] were added, and all the mixture was dissolved in 30 ml ethylene glycol and stirred for 60 min. After obtaining a homogeneous solution, it was transferred to a 50 ml Teflon-lined stainless autoclave and heated at 180 °C for 24 h. Finally, the precipitate was collected by centrifugation, washed with distilled water several times, and dried in air at 60 °C for 24 h to get the samples in powder form. The samples were named ZFO-1, ZFO-2, ZFO-3, and ZFO-4 for the sodium acetate concentrations 11.5, 20.5, 41.5, and 62.2 mmol, respectively.

### Materials characterization

X-ray diffractometer (XRD) with Cu K_α_ radiation of wavelength (λ = 1.54056Å) was used to identify the phase and crystal structure of ZnFe_2_O_4_ NPs at bulk scales. The experiment was conducted at room temperature, and the angle range was from 15 to 90° with an increment of 0.05. The average crystallite sizes of ZnFe_2_O_4_ NPs were calculated using the Williamson-Hall Equation^22^:

1 $$ \beta \cos \theta = \left( {{\raise0.7ex\hbox{${k\lambda }$} \!\mathord{\left/ {\vphantom {{k\lambda } D}}\right.\kern-0pt} \!\lower0.7ex\hbox{$D$}}} \right) + 4\varepsilon \sin \theta $$

where *k* is constant (0.94), *λ* is the X-ray wavelength, *β* is the full width at half maximum (FWHM), *θ* is the peak angle position, and *ɛ* is the strain. By plotting a graph between *βcosθ* on the y-axis and 4*sinθ* on the x-axis, the slope would give the strain, and the intercept would give the crystallite size. The average lattice constant (*a*) was calculated by plotting a graph between the lattice constants and *cos*^2^*θ/sinθ* at different peak positions using the equation:

2 $$ a = \left( {{\raise0.7ex\hbox{$\lambda $} \!\mathord{\left/ {\vphantom {\lambda {2\sin \theta }}}\right.\kern-0pt} \!\lower0.7ex\hbox{${2\sin \theta }$}}} \right)\sqrt {h^{2} + k^{2} + l^{2} } $$

The intercept of the straight line with the y-axis gives the accurate value of the lattice parameter for each sample^23^. A FESEM system of model Quanta 3D from ThermoFisher Scientific was used to investigate the surface morphology of synthesized materials. Before the analysis, the samples were gold-coated first to reduce charging. The accelerating voltage used was 5 kV during the imaging and 30 kV during the EDS analysis. The TEM analysis was carried out with a double aberration-corrected microscope of model Titan Themis Z 60–300. It was utilized to determine the nanoscale's structure, morphology, and elemental distribution. Compositional and chemical analysis were performed on ZnFe_2_O_4_ NPs using Escalab Xi + from ThermoFisher X-ray Photoelectron Spectroscopy (XPS) Scientific in macroscale and core-loss electron energy loss spectroscopy (EELS) in nanoscale. The specific surface area was obtained for the studied samples using Brunauer–Emmett–Teller (BET) with N_2_ as the adsorbate at liquid nitrogen temperature and Barrett–Joyner–Halenda (BJH) methods on a Micrometrics surface area analyzer. Bandgap measurement was carried out using a UV–visible diffused-reflectance Spectrometer. Tauc plot was used to calculate the bandgap using the Equation^24^:

3 $$ \left( {\alpha h\nu } \right)^{2} = A\left( {h\nu - E_{g} } \right) $$

where A is constant, hν is the photon energy, E_g_ is the bandgap, α is the absorption coefficient equals to:

4 $$ \alpha = {\raise0.7ex\hbox{${\left( {1 - R} \right)^{2} }$} \!\mathord{\left/ {\vphantom {{\left( {1 - R} \right)^{2} } {2R}}}\right.\kern-0pt} \!\lower0.7ex\hbox{${2R}$}} $$

and R is the reflectance. By plotting a graph between (αhν)^2^ on the y-axis and Energy (hν) on the x-axis, the intercept of the straight-line region with the x-axis would give the direct bandgap value. Finally, in-situ transmission electron microscopy was performed on the ZFO-3 sample to study ZnFe_2_O_4_ nanoparticles in the CO_2_ reduction process. The measurement was conducted using an Atmosphere™ holder from Protochips, Inc., with a 200 kV microscope of model Talos 200 from ThermoFisher Scientific. Argon gas was also utilized to compare CO_2_ reduction experiments with an inert gas. This technique is widely applied to characterize catalysts’ morphology and property changes in real-time by introducing light, heat signals, electricity, and gas during the measurement. Due to the feasibility of recording HRTEM images in sequence with SAEDs, the in-situ HRTEM imaging method proves fully adept at capturing and visualizing phase evolution. Moreover, this approach enables the direct correlation of phase dynamics with concurrent morphological alterations.

### Computational details

The first principal quantum mechanical calculations based on DFT were performed to obtain the electronic structures of (001) slab of ZnFe_2_O_4_ with a unit cell containing 56 atoms. Density of States (DOS) and Band Structure have been calculated by the Quantum ESPRESSO computational package^25^ using a plane wave set and pseudopotentials. The CIF file of ZnFe_2_O_4_ was extracted from “Materials Project,” and then (001) surface termination was performed on it. Generalized Gradient Approximation (GGA)^26^ with Perdew, Burke, and Ernzerhof (PBE) functions were used to describe the exchange–correlation interaction among the valence electrons. Broyden-Fletcher-Goldfarb-Shannon (BFGS) algorithm was used for geometry optimization^27^. Since we are dealing with magnetic material, it is preferred to use Projector Augmented Wave (PAW) pseudopotential, which could give more reliable results compared to Ultra Soft Pseudopotential (USPP). The Brillouin zone integration was carried out with a cold smearing technique. The k-point mech used for the calculations was 4 × 4 × 1, while the cut-off energy for wavefunction and charge density was set to be 75 Ry and 500 Ry, respectively.

## Results and discussions

### Structural and morphological analysis

**Figure 2:**
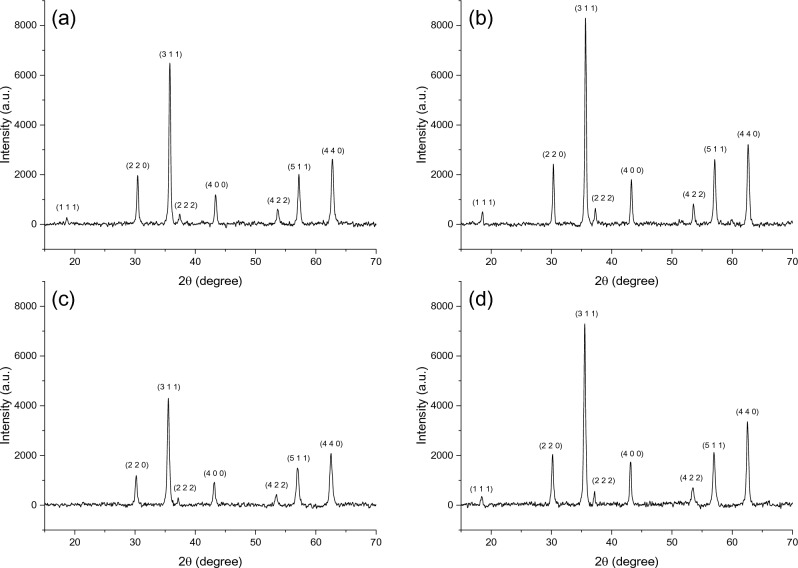
XRD Patterns of (**a**) ZFO-1, (**b**) ZFO-2, (**c**) ZFO-3, and (**d**) ZFO-4.

The structure of the synthesized samples at the bulk scale was first investigated using XRD. Figure 2 shows XRD patterns of all four ZnFe_2_O_4_ that had been synthesized with different concentrations of sodium acetate. The presented in Fig. 2 clearly shows that all samples have a single-phase structure without any extra peak resulting from unreacted precursors or other by-products. A slight peak shift was observed, reflecting a change in the lattice parameter of the synthesized samples. The lattice parameter was calculated from the intercept of a straight line with the y-axis, as shown in Fig. S1a. The broadening in diffraction peaks indicates the nanoscale of the crystallites. From the Williamson-Hall plot shown in Fig. S1b, the obtained crystallite size and strain were tabulated and are presented in Table 1. The obtained values of lattice parameters are very close to the literature^11,28^. It can also be noticed that crystallite size for all samples lies in the range of 20–35 nm, implying that the synthesized zinc ferrite particles are composed of nanoscale particles. The result obtained from the XRD experiment demonstrates the ability of sodium acetate to tune the structural properties of -ferrite material. On the other hand, the linear relationship in the Williamson-Hall plot has a positive gradient, which could be attributed to the presence of tensile strain for all samples^9^.

**Table 1:** Lattice parameter, crystallite size, and strain of ZnFe_2_O_4_ NPs.

Sample	Lattice parameter (Å)	Crystallite size (nm)	Strain (× 10^−3^)
ZFO-1	8.408	34.7	1.18
ZFO-2	8.41	43.3	1.27
ZFO-3	8.411	27.1	0.92
ZFO-4	8.407	29.7	0.97

**Figure 3:**
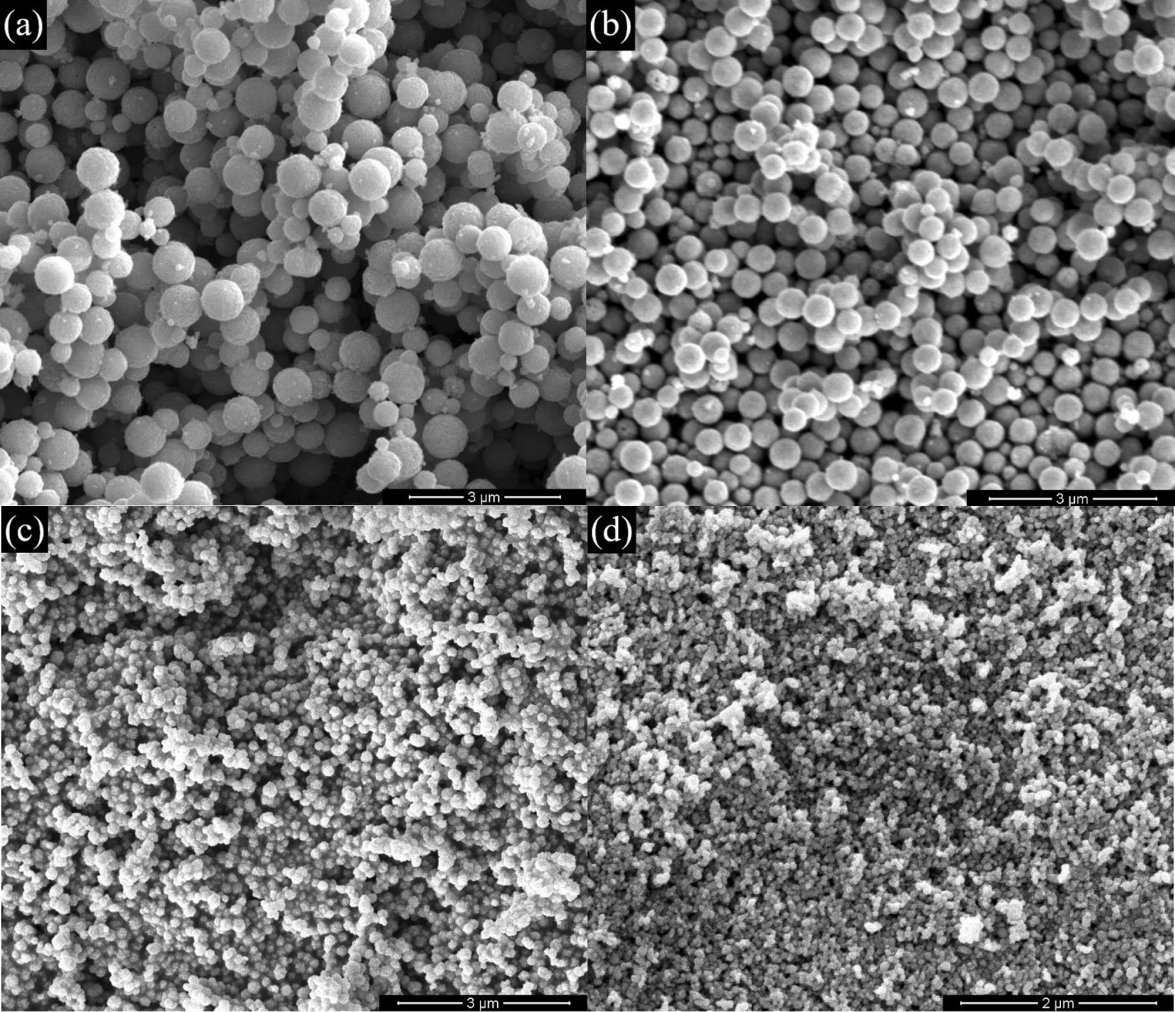
FESEM Images of (**a**) ZFO-1, (**b**) ZFO-2, (**c**) ZFO-3, and (**d**) ZFO-4.

The morphology and elemental composition of the synthesized ZnFe_2_O_4_ NPs were analyzed with FESEM, and the results are shown in Fig. 3. It can be noticed from Fig. 3 that most particles possessed spherical shapes and also tended to agglomeration, possibly due to the magnetic dipole moments interactions^29^. These nanospheres are formed by self-assembling small crystallites together, as these crystallites have 29–43 nm size, as learned from the XRD experiments. The average diameter of NPs is in the nm range and is followed by a gradual decrease with increasing the concentration of sodium acetate. The gradual reduction in the ZFO-1 (600 nm) grain sizes to ZFO-4 (50 nm) samples further proved that grain growth was slowed down. This finding reflects the effect of sodium acetate in size reduction, as it acts as an electrostatic stabilizing agent to prevent the accumulation of the primary magnetic nanoparticles in the reaction system^30^. It also acts as a protective reagent, meaning the products contain many spheres and nanoparticles^31^.

**Figure 4:**
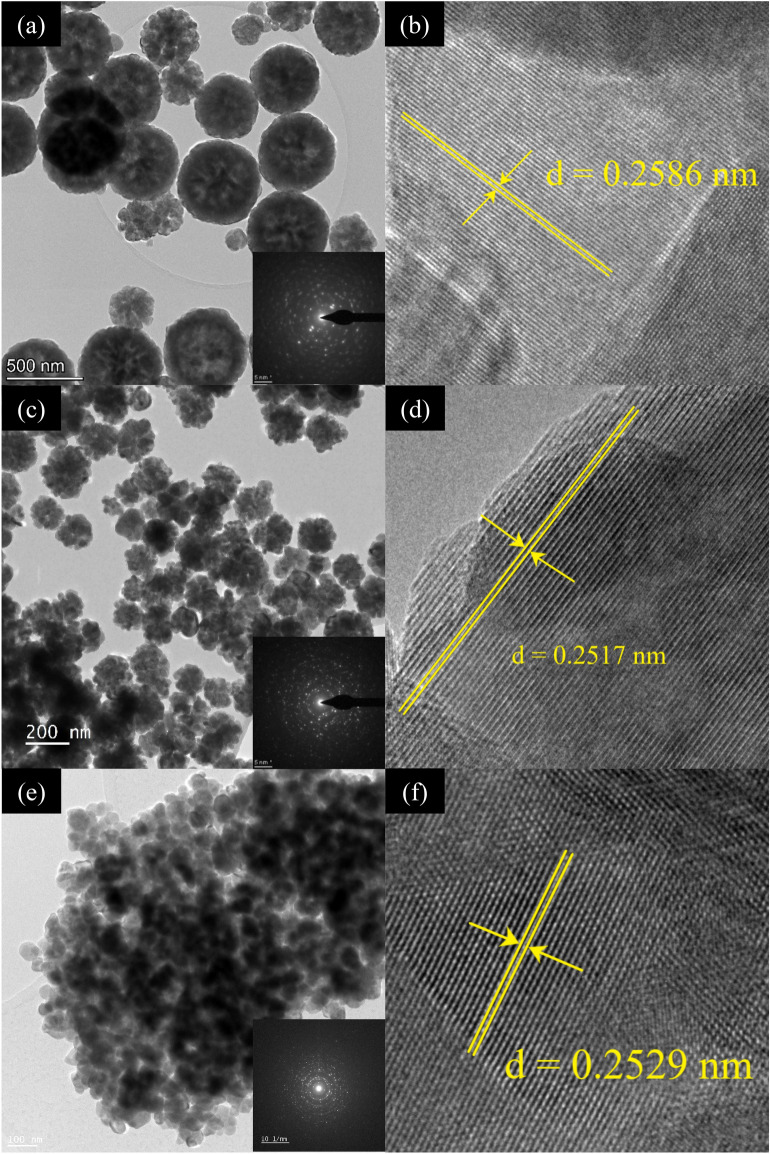
TEM and HRTEM images of (**a**, **b**) ZFO-2, (**c**, **d**) ZFO-3, and (**e**, **f**) ZFO-4, respectively.

Figure 4 shows the TEM images of samples ZFO-2, ZFO-3, and ZFO-4. As expected, the particles of all samples have nano-spherical morphology with an average diameter from 600 to 50 nm. It is in good agreement with the results obtained with FESEM analysis. Again, particle size showed a gradual decrease with increasing sodium acetate amount. Comparing our values with the literature that used the same fuel, it was found that our spherical particles are finer and have lower diameter^31,32^. It is also apparent that the accumulation of nanoparticles increases with the reduction of their size. Again, we believe accumulation occurs due to the magnetic interaction and weak Van-der Wall force between the nanoparticles. It is also evident that this accumulation of NPs increases with their decreasing size. Images of ZFO-2 and ZFO-3 reveal that the nanospheres formation is again due to the assembling of small crystallites with very fine sizes. The HRTEM images in Fig. 4 show interference fringes with spacings in the range of 0.260 nm, 0.252 nm, and 0.253 nm. These spacings represent the interplanar spacings of (3 1 1) planes of face-centered cubic ZFO-2, ZFO-3, and ZFO-4, respectively. In addition, the inset shown in the exact figure demonstrates the SAED of the studied samples, which confirms the polycrystalline characteristic associated with the (1 1 1), (2 2 0), and (3 3 1) planes of the FCC ZnFe_2_O_4_. These results confirm the previously discussed XRD results.

### Compositional and chemical analysis

**Figure 5:**
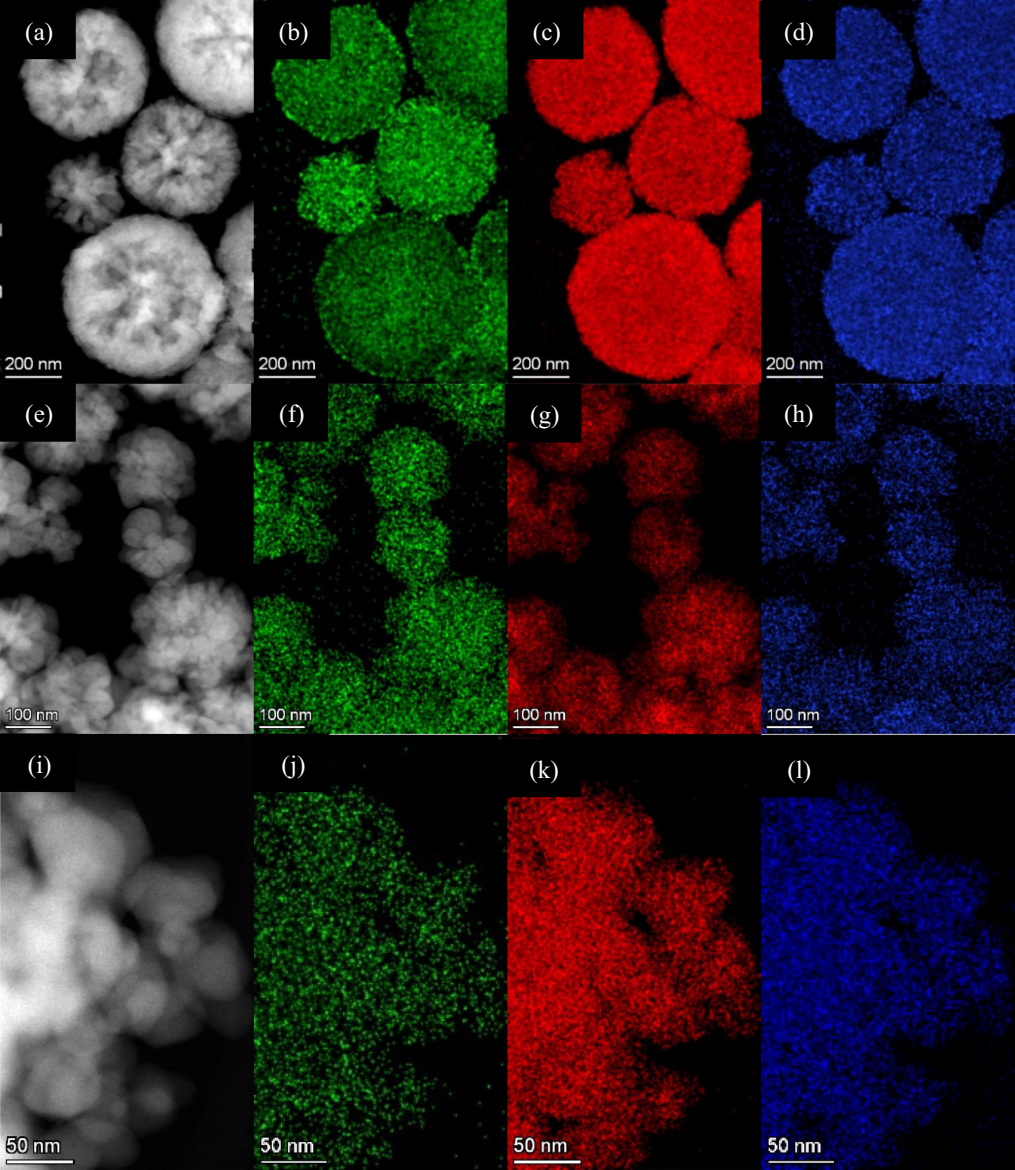
TEM-EDS elemental mapping of (**a**–**d**) ZFO-2, (**e**–**h**) ZFO-3, and (**i**–**l**) ZFO-4.

TEM-EDS elemental analysis was performed to confirm the purity of the synthesized samples, and the results are shown in Fig. S2. The presence of Zn, Fe, and O peaks for all samples without any additional element except the peak at 8.15 keV, the Cu-K peak from the Cu grid. The overall results obtained from TEM-EDS reveal a high purity of the samples. Table S1 in the SI file shows the variation of atomic percent for each element with reducing particle size (i.e., increasing sodium acetate concentration). The spatial distribution of each component with the particles was investigated using the STEM-EDS spectrum imaging (SI) method, and the obtained elemental maps of elements are shown in Fig. 5. The uniform distribution was observed in the generated map of each element. Each sample's black and white image represents the High Angle Annular Dark Field (HAADF) imaging, while the green, red, and blue colors represent Zn, Fe, and O elements, respectively. The lower brightness of the Zn image for ZFO-4 than the other samples explains the decrease of its atomic percent, as shown in the TEM-EDS elemental analysis in Table S1. The HAADF images again reveal the tendency of nanoparticles to agglomerate as the particle size decreases.

**Figure 6:**
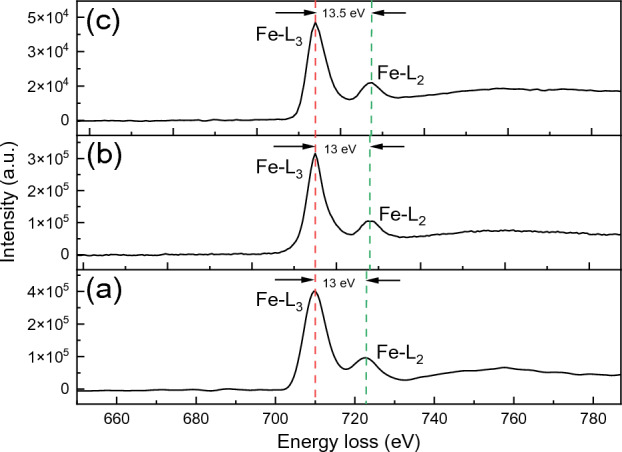
Fe-L_23_ energy loss EELS edges in (**a**) ZFO-2, (**b**) ZFO-3, and (**c**) ZFO-4.

Further elemental analysis was performed on the nanoscale using the EELS spectrum. Figure 6 shows the EELS spectra of Fe-L_2,3_ edges for ZFO-2, ZFO-3, and ZFO-4. Table S1 demonstrates the elemental quantification from EELS spectra as well. The atomic percent from EELS spectra is very close to those of TEM-EDS analysis. The range of intensities of all edges for ZFO-4 are lower than ZFO-2 and ZFO-3. The lower peak intensity in EELS spectra means a higher concentration of oxygen vacancies^33,34^. So, we can find from this explanation that the ZFO-4 sample with the smallest particle size has more oxygen vacancies than the other two samples. These results confirm that reducing particle size could increase the formation of oxygen vacancies. It is believed that the oxygen vacancies pave a path to enhance the capacity of metal oxides for supercapacitors and other applications such as electrocatalysis and battery^35^. On the other hand, the relative intensities between L_2_ and L_3_ and chemical shifts for Fe edges could provide us with the ionization state of Fe ions^34^. The intensity ratio L_3_/L_2_ for ZFO-2, ZFO-3, and ZFO-4 samples was 3.85, 3.76, and 3.56 respectively. Besides, the separations between the L_3_ and L_2_ lines are 13 and 13.3 eV for ZFO-3 and ZFO-4, respectively. The change in intensity ratio and chemical shift indicates a change in the oxidation state from Fe^3+^ to Fe^2+^^36^. The valence of Fe is completely 3+ when the intensity ratio value reaches 5.5^37^. By comparing this reference value with our values, we will find that 30%, 31.6%, and 35.3% of Fe^3+^ converted to Fe^2+^ for ZFO-2, ZFO-3, and ZFO-4, respectively. This result indicates that reducing the size by increasing the sodium acetate can partially convert the Fe^3+^ to Fe^2+^ on the sample surface. Our intensity ratio values were less than in the literature^36^, indicating a higher probability of Fe^2+^ formation on the surface of the studied samples due to the size effect.

**Figure 7:**
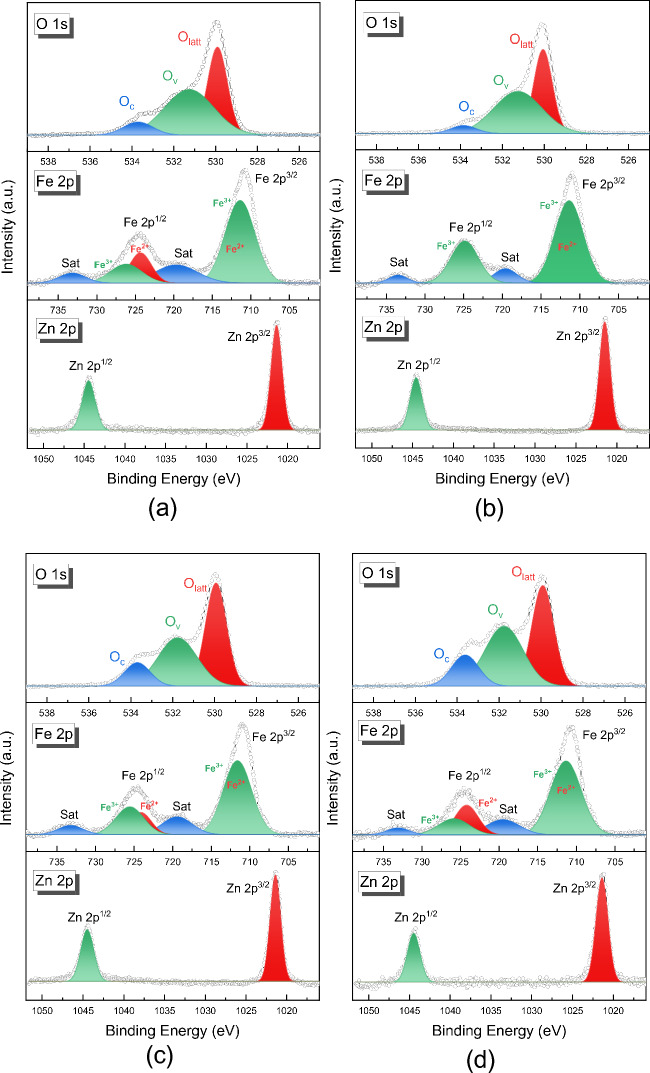
XPS core-level spectra of the ZnFe_2_O_4_ derived from (**a**) ZFO-1, (**b**) ZFO-2, (**c**) ZFO-3, and (**d**) ZFO-4.

For the characterization of the elemental composition as well as the chemical state, XPS was performed for all samples. The XPS survey is shown in Fig. S3, attached to the supplementary information file. The high-resolution XPS spectra corresponding to the core levels of ZnFe_2_O_4_ derived from ZFO-1, ZFO-2, ZFO-3, and ZFO-4 are shown in Fig. 7a–d, respectively. The Zn 2p core levels show the spin–orbit splitting of Zn 2*p*^1/2^ and Zn 2*p*^3/2^ core level states around ~ 1044 and 1021 eV, respectively. This result confirms the existence of Zn^2+^ in the tetrahedral sites for the three samples^38^. In addition, no shoulder peaks appear, meaning there are no Zn cations in the octahedral site^9^. The high-resolution spectrum of the Fe 2*p* core levels shows double peaks at ~ 725 and 711 eV, corresponding to Fe 2*p*^1/2^ and Fe 2*p*^3/2^, respectively. Both contributions are somewhat asymmetric, and there are more than one Fe species in the near-surface region of the ZnFe_2_O_4_ particles^39^. To confirm this, the Fe 2*p*^3/2^ peak was deconvoluted into two peaks with binding energies at ~ 710.5 and 711.5 eV corresponding to Fe^2+^ in octahedral sites and Fe^3+^ in tetrahedral sites, respectively^40^. Similarly, the peak Fe 2*p*^1/2^ was deconvoluted to two peaks at ~ 724 and 726 eV, corresponding to Fe^2+^ and Fe^3+^, respectively. Moreover, at ~ 733 and 719 eV, a broad contribution represents the satellite peaks of the Fe 2*p*^1/2^ and Fe 2*p*^3/2^, respectively, indicating the presence of Fe^2+^ in the sample^39^, as discussed above in the EELS analysis. In the high-resolution O 1s core level, the signals also exhibit asymmetric peaks, and their broadening indicates that there are multiple oxygen species. O 1s core level XPS spectrum was fitted into three peaks to confirm this. The peaks at ~ 530, 531, and 534 eV can be associated with the O^2−^ ions of the lattice oxygen of ZnFe_2_O_4_ (O_latt_), oxygen vacancies in the lattice (O_v_), and surface absorbed oxygen-containing species (O_c_), respectively^41^.

### Surface area and pore size analysis

**Figure 8:**
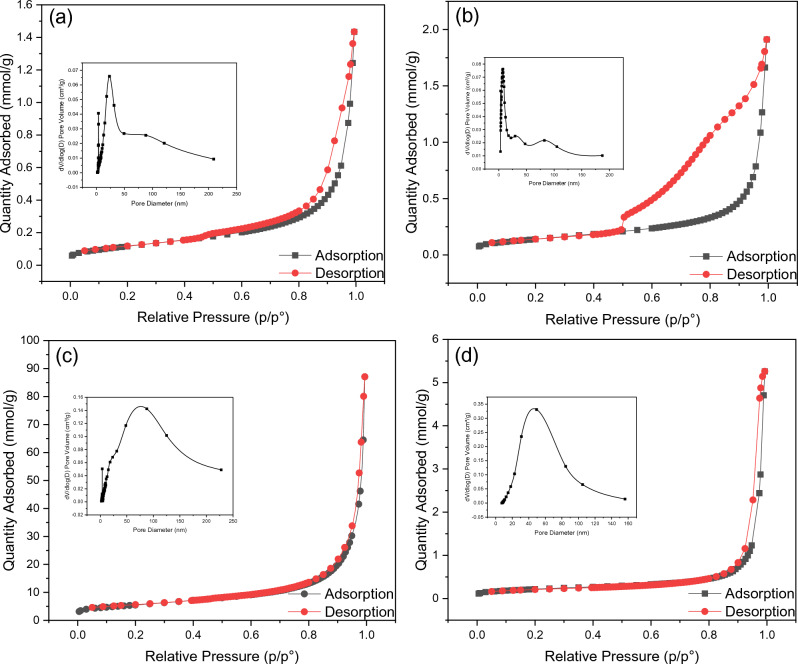
N_2_ adsorption–desorption analysis of (**a**) ZFO-1, (**b**) ZFO-2, (**c**) ZFO-3, and (**d**) ZFO-4.

Due to the importance of specific area and porosity for several applications, N_2_ adsorption–desorption measurements were performed to calculate the specific surface area and the pore size of ZnFe_2_O_4_ NPs, and the obtained results are shown in Fig. 8. All samples showed an IV-type isotherm profile, which is associated with capillary condensation taking place in mesopores^42^. There are four types of hysteresis, depending on their shape. The shape we obtained from the studied samples here is an H1 type. This type is often associated with porous materials known, from other evidence, to consist of agglomerates or compacts of approximately uniform spheres in regular arrays and, hence, to have narrow pore size distributions^42^. These results match what we obtained from FESEM and TEM investigations. Table 2 contains the investigated samples’ surface area, particle size, pore size, and volume values. The results showed that the surface area varied from 9 to 19 m^2^/g and was found to have an increasing trend with increasing sodium acetate amount. As mentioned earlier, the rising sodium acetate amount decreases the particle size, and therefore, it makes sense that the surface area increases. A larger surface area provides more active sites, promoting gas diffusion during sensing^15,43^. The pore-size distribution of the samples was calculated using the Barrett–Joyner–Halendam (BJH) method and was plotted in the inset of Fig. 8. It can be noticed from therein that the ZnFe_2_O_4_ samples formed a porous structure with a wide range of pore size distributions from 1 to 100 nm. For the pore volume distribution, it also shows a gradual increase with reducing particle size. That makes sense because the pore volume depends on the pore size, which also gradually increases. The pore volume (P_v_) could be correlated to the pore size (P_s_) and surface area (S_a_) by using the Wheeling Equation^44^:

5 $$ P_{s} \left( {nm} \right) = 4 \times 10^{3} \frac{{P_{v} \left( {cm^{2} /g} \right)}}{{S_{a} \left( {m^{2} /g} \right)}}. $$

**Table 2:** Surface area, pore volume, pore size, and particle size of ZnFe_2_O_4_ NPs.

Sample	Surface area (m^2^/g)	Pore volume (cm^3^/g)	Pore size (nm)	Particle size (nm)
ZFO-1	9.63	0.047829	19.87	600
ZFO-2	11.51	0.063975	22.23	400
ZFO-3	19.5	0.124181	25.46	150
ZFO-4	17.31	0.181391	41.91	50

### Bandgap measurement

**Figure 9:**
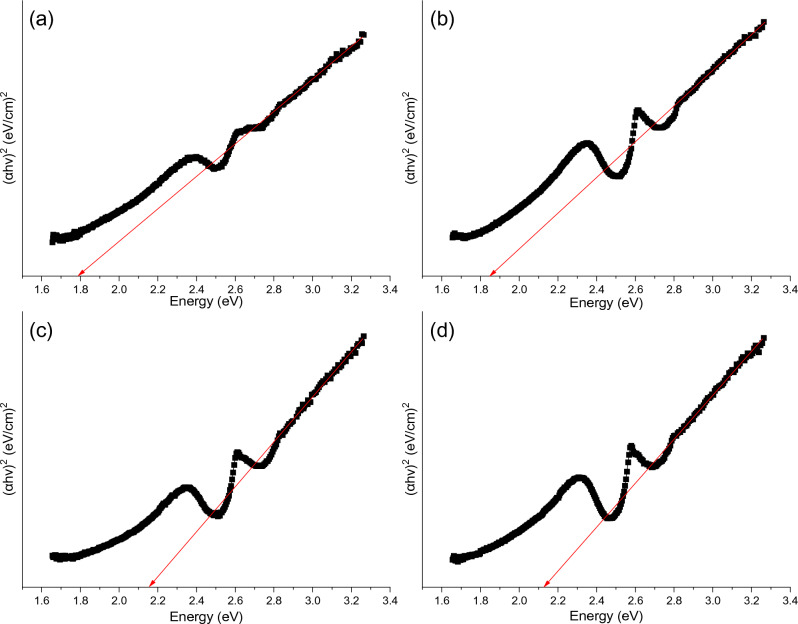
Tauc’s plot for the bandgap determination of (**a**) ZFO-1, (**b**) ZFO-2, (**c**) ZFO-3, and (**d**) ZFO-4.

Bandgap was calculated experimentally for all studied samples from Fig. 9. The intercept of the straight-line region with the x-axis could give the bandgap value. Bandgap values obtained from the plots are 1.79, 1.85, 2.15, and 2.13 for ZFO-1, ZFO-2, ZFO-3, and ZFO-4, respectively. These values exceed their corresponding ZnFe_2_O_4_ bulk value (1.67 eV). Besides, increasing the sodium acetate amount could tune the bandgap value by reducing the particle size due to the quantum confinement effect. These values closely agree with Shaterian et al., who suggested that these materials with such bandgaps would benefit photocatalysis activities and photodegradation due to their excellent adsorption in visible regions^30^.

### DFT study

**Figure 10:**
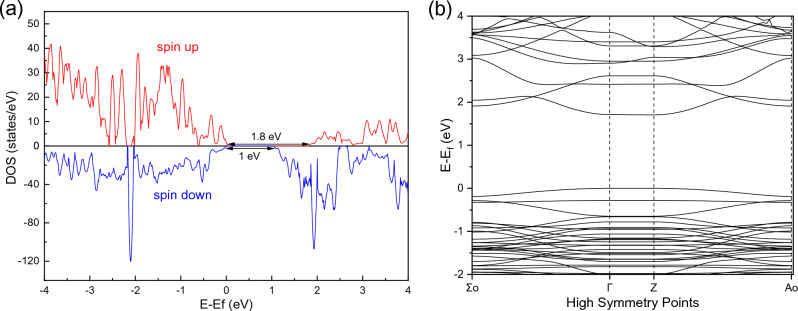
(**a**) Total density of states and (**b**) band structure of ZnFe_2_O_4_ (001) surface terminated.

The electronic structure of zinc ferrite nanoparticles in samples was determined by performing the first principal calculations using DFT. The quantum ESPRESSO package was used for DFT calculations because of its ability to generate results with a higher degree of accuracy. To calculate the electronic properties of ZnFe_2_O_4_ on the nanoscale, a surface termination at the (001) plane was done, as observed in the HRTEM analysis of the samples. The file was then relaxed after extracting the CIF file of ZnFe2O4 and making (001) surface termination for it using VESTA software. Figure S4 shows the unit cell before and after the surface termination. The original lattice parameter of the ZnFe_2_O_4_ is a = b = c = 8.3448 Å, while after (001) surface termination, it became a = b = 7.9450 Å and c = 18.6473 Å. Then, the relaxed values of lattice parameters and atomic positions were put in the Self and Non-Self-Consistent Filed (SCF and NSCF) calculations. The SCF was used to calculate the band structure, and NSCF was used to calculate the total Density of States (DOS). The DOS of ZnFe_2_O_4_ computed from DFT is shown in Fig. 10a. It is obvious that due to the existence of two spin states, there are DOS for spin up and DOS for spin down. There is a difference in the bandgap value between spin up and down (1.8 eV and 1 eV, respectively). The band calculation could give the exact bandgap value. As shown in Fig. 10b, it is evident that ZnFe_2_O_4_ has a direct bandgap across the Gamma point of about 1.71 eV, which is very close to our obtained experimental value for ZFO-1 (1.79 eV). This slight underestimation is attributed to the limitation of DFT calculations. On the other hand, the obtained bandgap value is more significant than that of the bulk one (1.67 eV). This could be attributed to the quantum confinement for material at the nanoscale, which causes an increase in the band gap. Upon comparative analysis of the acquired bandgap value with experimental data, it becomes evident that remarkable proximity exists between the two, thereby bolstering the validity of our assumptions regarding the Density Functional Theory (DFT) calculations.

### In-situ TEM measurement

**Figure 11:**
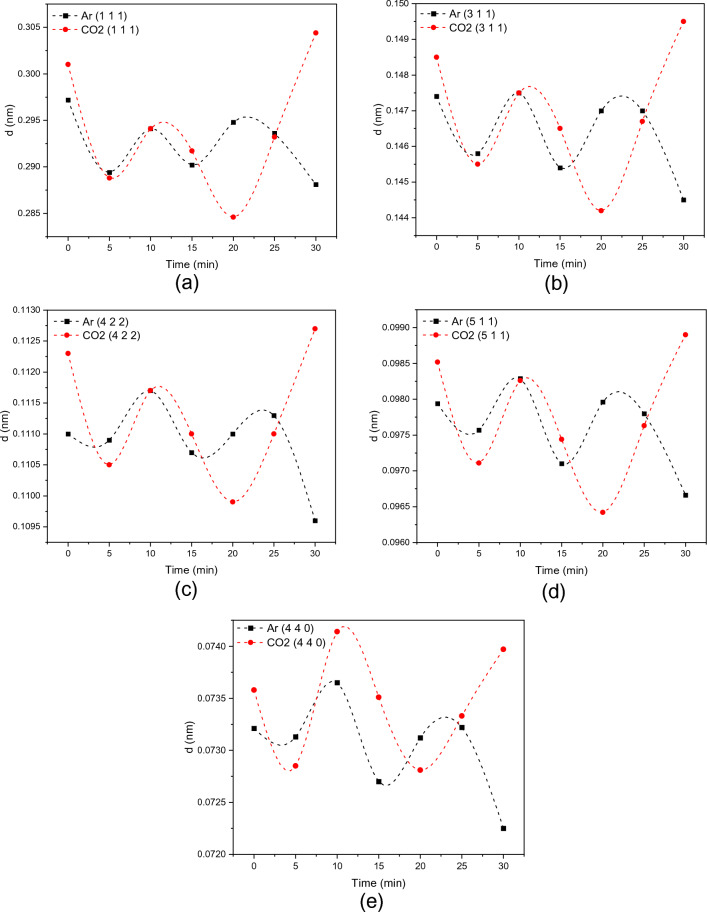
Variation of the d-spacing of ZFO-3 with time during purging Ar and CO_2_ for (**a**) (1 1 1), (**b**) (3 1 1), (**c**) (4 2 2), (**d**) (5 1 1), and (**e**) (4 4 0).

In pursuing this objective, we employ the in situ dry cell transmission electron microscopy (TEM) methodology, enabling immediate and direct visual analysis of nanoscale and atomic-level structural modifications as they occur in real time. In this work, we investigate the reaction of the ZFO-3 sample with ~ 100 nm particle size with the Ar and CO_2_ at 300 °C operating temperature. Figures S5 and S6 in SI shows the TEM images and SAED of ZFO-3 when exposed to Ar and CO_2_ for 30 min and an interval of 5 min, respectively. The images showed no noticeable change during exposure to Ar and CO_2_ gases. However, the diffraction spots could show a slight change in intensity and shift. To confirm this, five points of the diffraction spots representing (1 1 1), (3 1 1), (4 2 2), (5 1 1), and (4 4 0) planes were chosen from all SAED patterns and analyzed using Digital Micrograph software to calculate their d-spacing and see how it would change. Figure 11 shows how the d-spacing changes when ZnFe_2_O_4_ NPs are exposed to Ar and CO_2_. The reason for using Ar is that it is an inert gas, and its existence would develop a good comparison with the CO_2_ nano-inert gas. When the material is exposed to Ar and CO_2_, it fluctuates. Both gases followed the same fluctuation with increasing reaction time except after 20 and 30 min. Knowing that the lattice parameter depends on the d-spacing since the h k l will not change, this fluctuation would alter the lattice parameter, i.e., make the crystal expand and shrink. This result reflects the capability of ZnFe_2_O_4_ NPs to absorb Ar and CO_2_ gases, especially after 20 and 30 min. At these specific times, the d-spacing changed significantly by ~ 5.4% in the (1 1 1) plane. This most likely concerns the kinetics of the Ar and CO_2_ interaction with the ferrite surface at 300 °C. Under Ar, shrinkage happens due to the annealing of the ferrite (under Ar, maybe oxygen is removed). Under CO_2_: CO_2_ is filling the vacancies (opposite to what happens under Ar)^45^. ZnFe_2_O_4_ has been identified as an n-type semiconductor^46^. In principle, when subjecting the ZnFe_2_O_4_ to elevated temperatures while exposed to air, active oxygen species are absorbed onto the surface of ZnFe_2_O_4_ NPs. This process involves physisorption of O_2_ molecules (${\text{O}}_{2}^{ - }$) at lower temperatures (< 200 °C) followed by chemisorption (O^−^ and O^2−^) at higher temperatures (> 200 °C), wherein mobile electrons (e^−^) are captured from the surface^47^. Consequently, a charge depletion layer forms on the surface of ZnFe_2_O_4_ NPs due to the hopping between Fe^3+^ and Fe^2+^ ions. Subsequently, different concentrations of CO_2_ gas are introduced to the surface, wherein CO_2_, recognized as an electron-withdrawing molecule, further extracts mobile electrons (e^−^) from the surface or interacts with the chemisorbed oxygen species. Consequently, this interaction leads to a reduction in the density of the primary charge carriers (e^−^) of the ferrite as described in the equations below:

6 $$ {\text{CO}}_{{2{ }\left( {\text{g}} \right)}} + {\text{O}}_{{\left( {{\text{ad}}} \right)}}^{ - } \to {\text{CO}}_{{2{ }\left( {{\text{ad}}} \right)}}^{ - } + {\raise0.7ex\hbox{$1$} \!\mathord{\left/ {\vphantom {1 2}}\right.\kern-0pt} \!\lower0.7ex\hbox{$2$}}{\text{O}}_{{2{ }\left( {\text{g}} \right)}} $$

7 $$ {\text{CO}}_{{2{ }\left( {\text{g}} \right)}} + {\text{O}}_{{\left( {{\text{ad}}} \right)}}^{2 - } \to {\text{CO}}_{{2{ }\left( {{\text{ad}}} \right)}}^{ - } + {\text{O}}_{{\left( {{\text{ad}}} \right)}}^{ - } $$

8 $$ {\text{CO}}_{{2{ }\left( {\text{g}} \right)}} + {\text{e}}^{ - } \to {\text{CO}}_{{2{ }\left( {{\text{ad}}} \right)}}^{ - } \to {\text{CO}}_{{\left( {\text{g}} \right)}} + {\text{O}}_{{\left( {{\text{ad}}} \right)}}^{ - } $$

**Figure 12:**
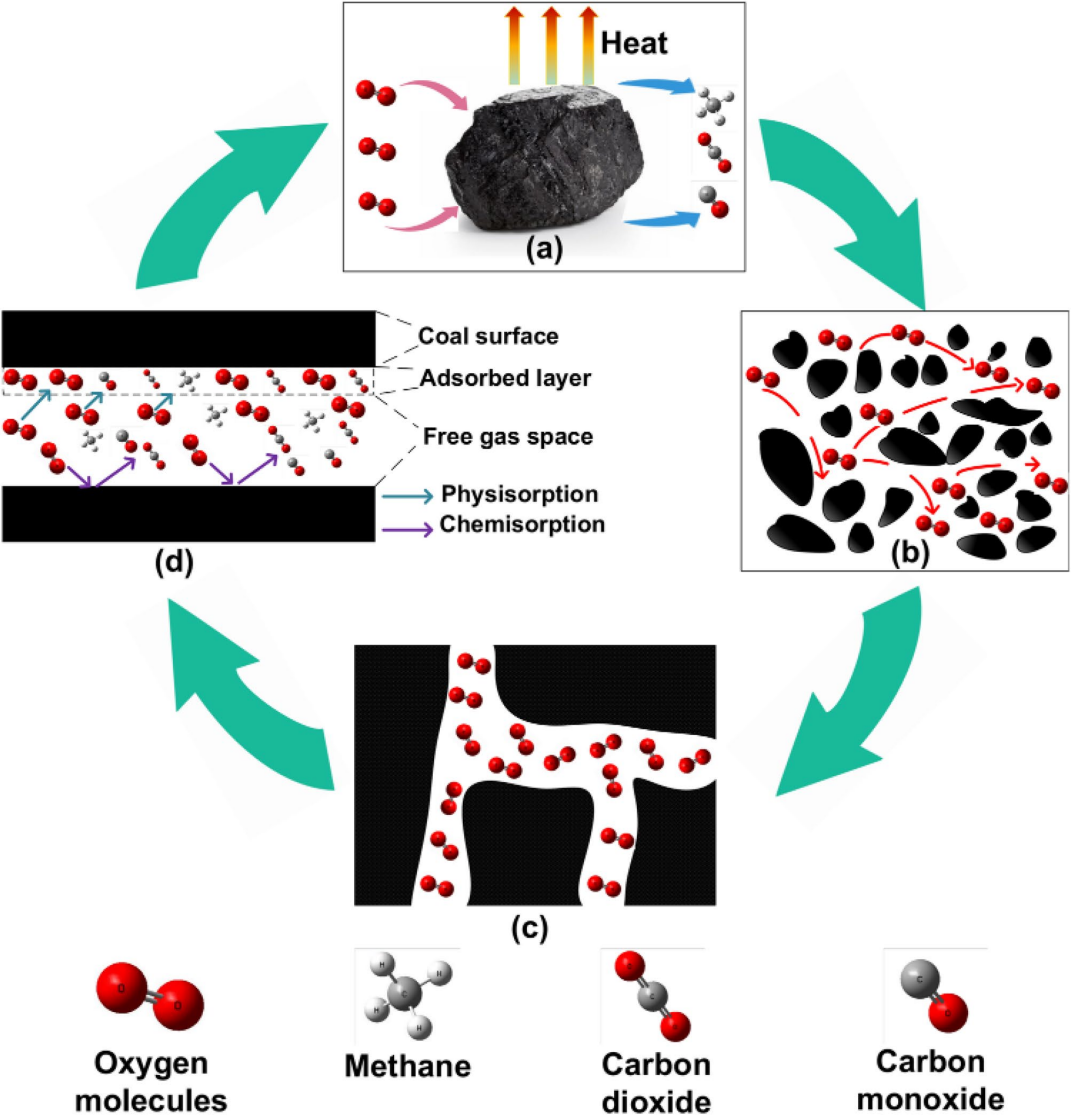
Schematic for the mechanism of gas interaction with ZnFe_2_O_4_ NPs.

Therefore, the observed expansion and shrinking of the d-spacings with the exposure of samples to gases may be attributed to the redox of the studied metal oxide material. This mechanism could be proposed as shown in Fig. 12.

## Conclusions

A facile hydrothermal method for the synthesis of ZnFe_2_O_4_ nanomaterial is developed. It is a versatile method that enables selecting a wide range of the sizes of nanoparticles in synthesized samples. A multi-faceted characterization approach for understanding the physical and chemical properties of the synthesized samples is crucial. In this case, it revealed that the presented hydrothermal method results in ZnFe_2_O_4_ nanomaterial with high purity, excellent crystallinity, (001) facets, and uniform elemental distribution of each element. The role of fuel turned out to be the most critical in controlling the size of particles in synthesized samples. Further, it prevents the accumulation of the primary magnetic nanoparticles in the reaction system, as it could act as an electrostatic stabilizing agent. The EELS and XPS analyses are crucial to determining the fraction of Fe^3+^ ions converted to Fe^2+^. The DFT calculations of total DOS and band structure are essential to confirm the size and type of the bandgap of synthesized ZnFe_2_O_4_ samples and showed a good agreement with the experimental values obtained from the UV-visible spectrometer. ZnFe_2_O_4_ NPs demonstrated a capability to adsorb CO_2_ gas, confirmed by the lattice parameter's expansion and shrinking. This makes this material with such size promising for detecting carbon dioxide and converting it to carbon monoxide.

### Supplementary Information

Supplementary Information.

## Data Availability

The datasets used and/or analyzed during the current study are available from the corresponding author on reasonable request.
