# Supplementary material for: Synthesis, characterization, and preliminary insights of ZnFe2O4 nanoparticles into potential applications, with a focus on gas sensing
Source: Sci Rep. 2023 Nov 11;13:19705. doi: 10.1038/s41598-023-46960-w (PMC10640627; doi:10.1038/s41598-023-46960-w)
Supplement: Supplementary file 1 — Supplementary Information. [file 41598_2023_46960_MOESM1_ESM.docx]

# Supplementary information to Synthesis, Characterization, and Preliminary Insights of ZnFe_2_O_4_ Nanoparticles into Potential Applications, with a Focus on Gas Sensing

Zeyad M. Abdulhamid^1^, Aasif A. Dabbawala^2^, Thomas Delclos^3^, Rainer Straubinger^4^, Magnus Rueping^5^, Kyriaki Polychronopoulou^2^, Dalaver H. Anjum^1,*^

^1^Department of Physics, Khalifa University of Science and Technology, Abu Dhabi 127788, United Arab Emirates; Center for Catalysis and Separations (CeCaS Center), Khalifa University of Science and Technology, Abu Dhabi 127788, United Arab Emirates.

^2^Department of Mechanical Engineering, Khalifa University of Science and Technology, Abu Dhabi 127788, United Arab Emirates; Center for Catalysis and Separations (CeCaS Center), Khalifa University of Science and Technology, Abu Dhabi 127788, United Arab Emirates.

^3^Manager, Materials, and Surface Core Labs, Khalifa University of Science and Technology, Abu Dhabi 127788, United Arab Emirates

^4^Core Technology Platforms, New York University Abu Dhabi, Abu Dhabi 129188, United Arab Emirates.

^5^KAUST Catalysis Center (KCC), King Abdullah University of Science and Technology, Thuwal 23955-6900, Saudi Arabia.

^*^Corresponding author email: [dalaver.anjum@ku.ac.ae](mailto:dalaver.anjum@ku.ac.ae)

Figure S1 Variation of lattice parameter with cos^2^θ/sinθ, and Williamson-Hall plots of (a)ZFO-1, (b) ZFO-2, (c) ZFO-3, and (d) ZFO-4, respectively.

Figure S2 TEM-EDS elemental analysis of ZnFe_2_O_4_ derived from (a) ZFO-2, (b) ZFO-3, and (c) ZFO-4.

Figure S3 XPS survey of the ZnFe_2_O_4_ derived from (a) ZFO-1, (b) ZFO-2, (c) ZFO-3, and (d) ZFO-4.


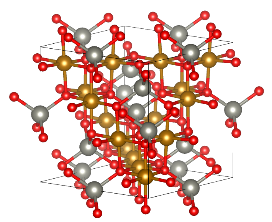

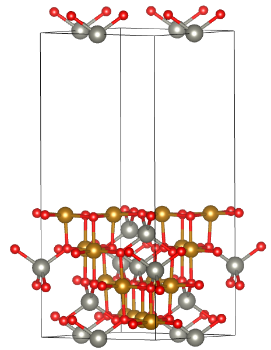

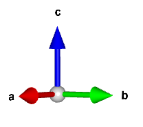


(b)

(a)

Figure S4 Unit cell of ZnFe_2_O_4_ (a) without and (b) with (001) surface termination.


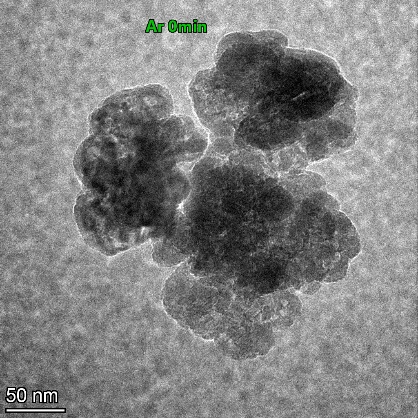

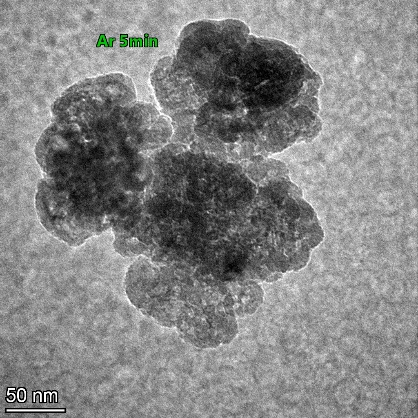

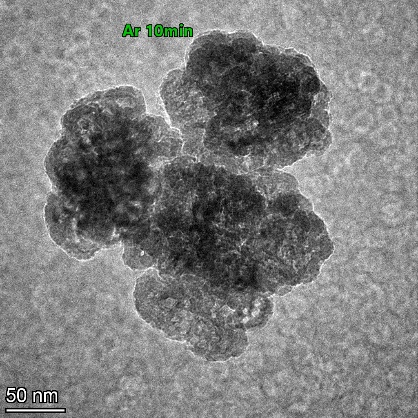

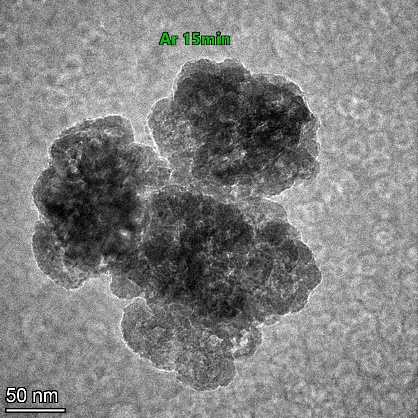

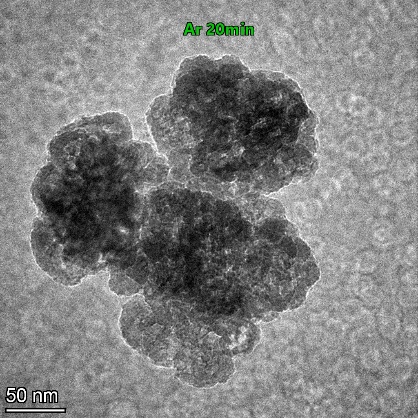

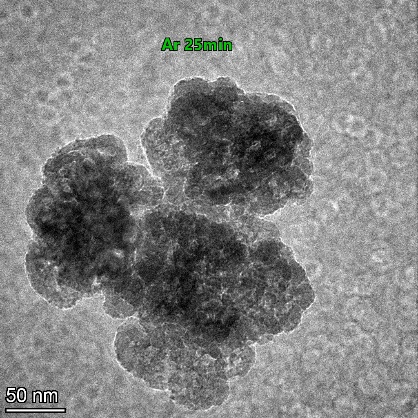

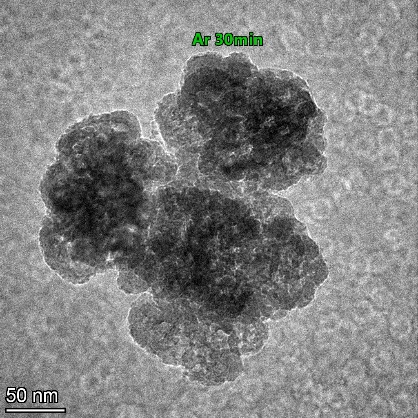


(a)


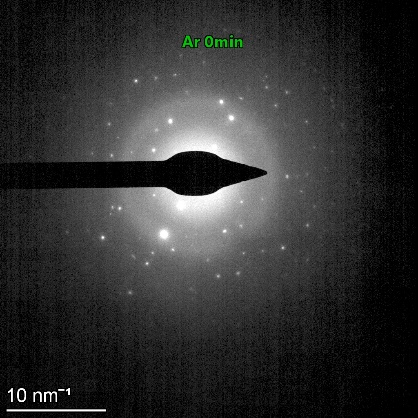

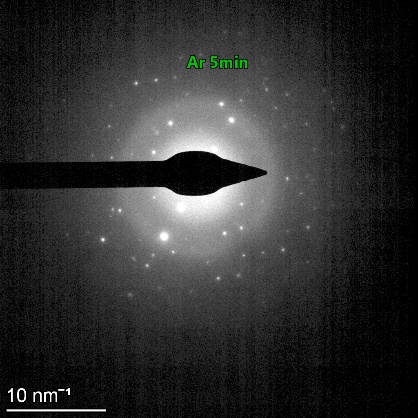

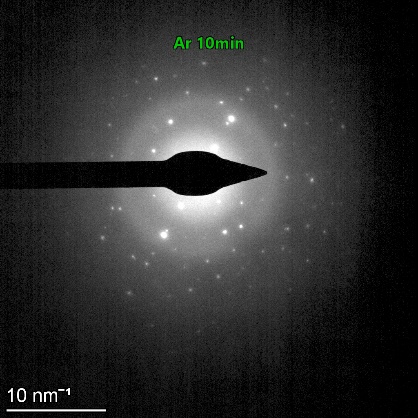

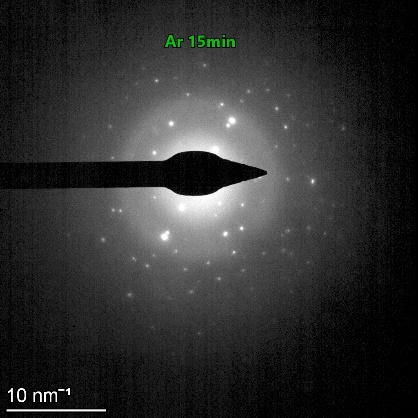

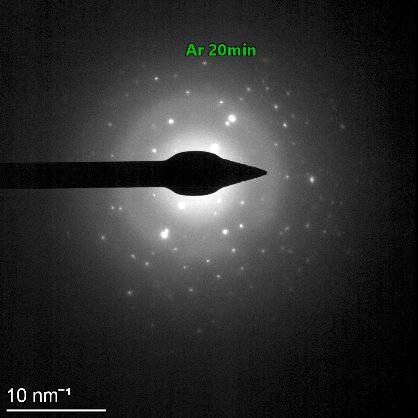

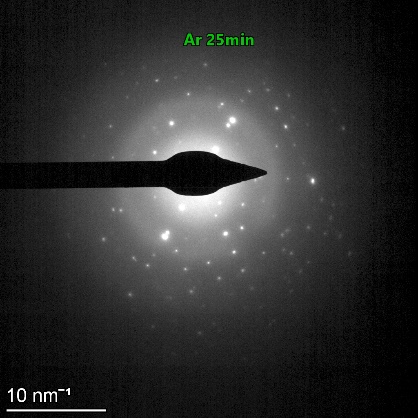

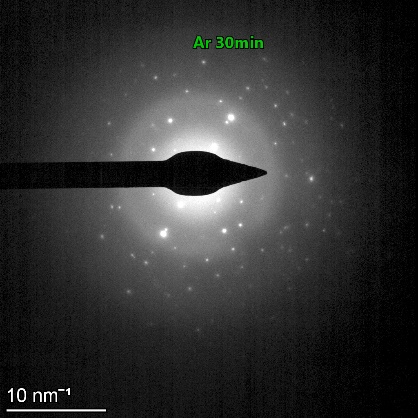


(b)

Figure S5 (a) TEM images and (b)SAED of ZFO-3 during purging Argon gas for 30 minutes at 300 ^o^C.


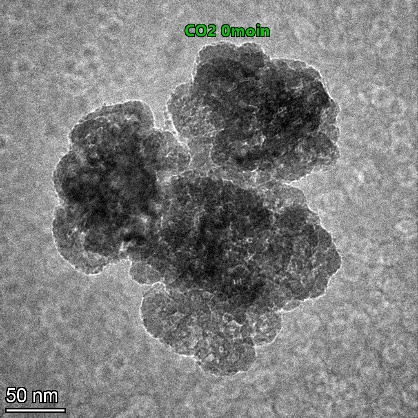

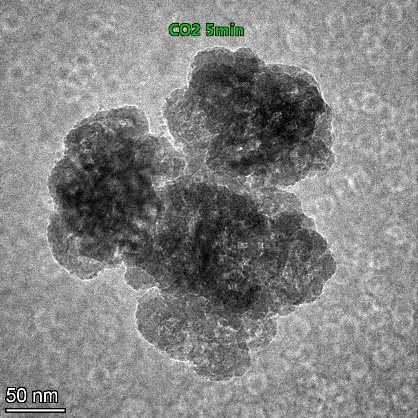

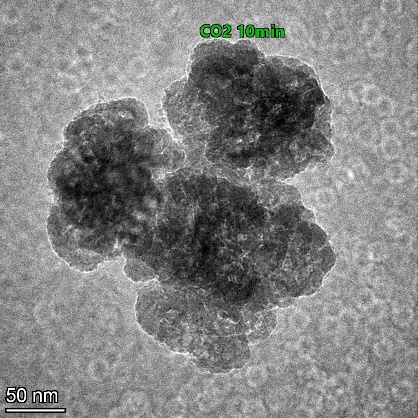

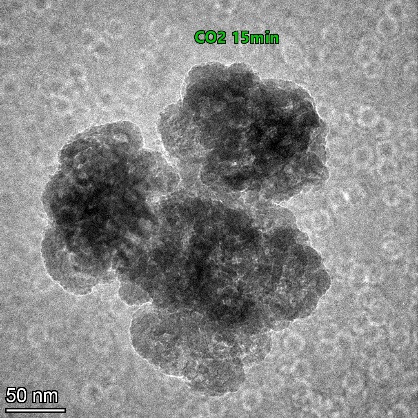

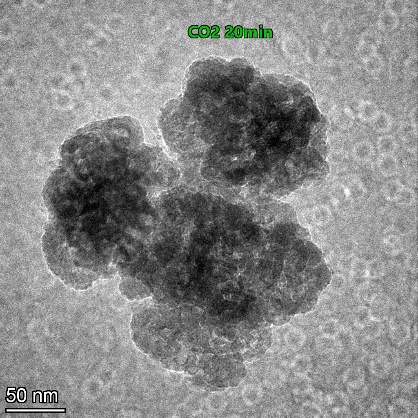

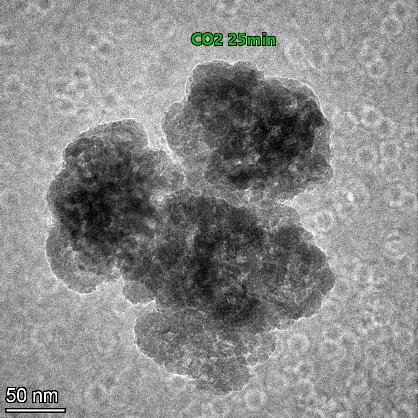

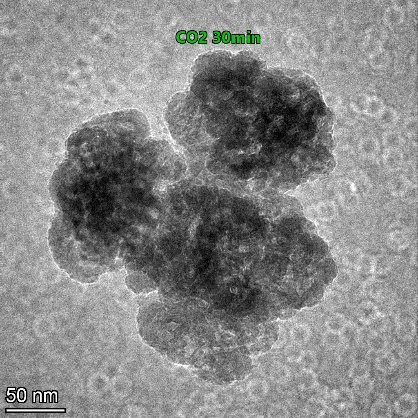


(a)


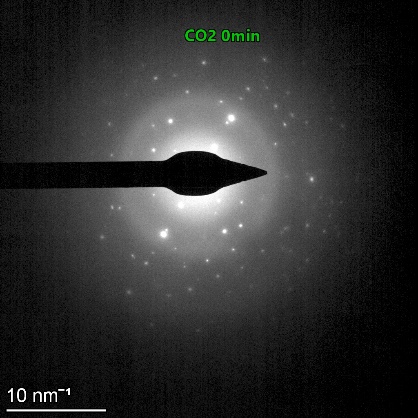

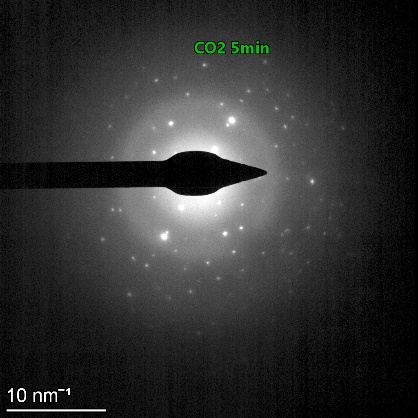

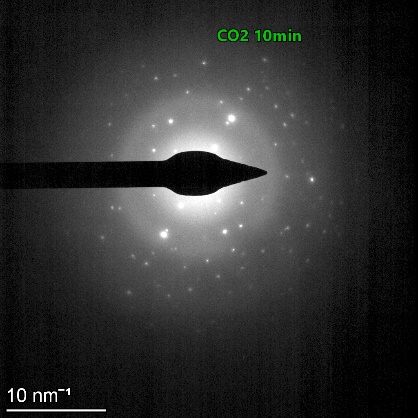

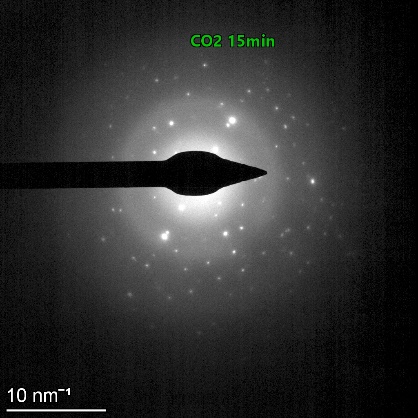

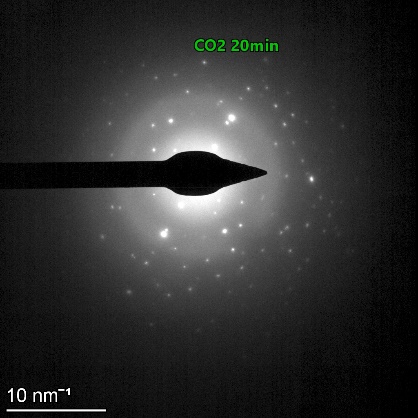

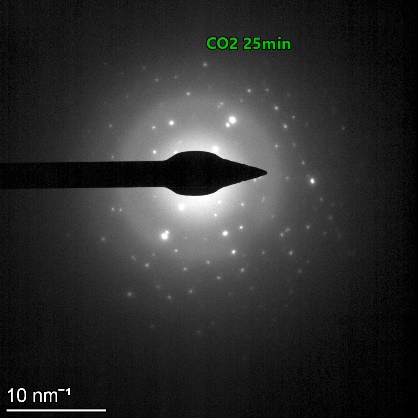

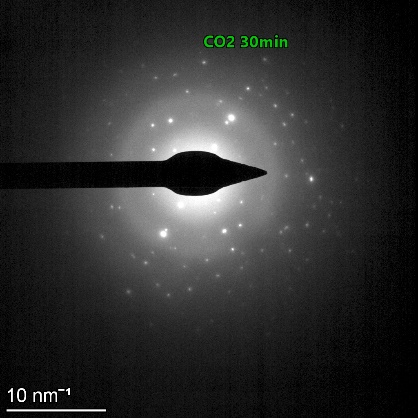


(b)

Figure S6 (a) TEM images and (b)SAED of ZFO-3 during purging CO2 gas for 30 minutes at 300 ^o^C.

Table S1 TEM-EDS and EELS elemental composition of ZnFe_2_O_4_ NPs.

| **Elements** | **Atomic Percent** | | | | | |
| --- | --- | --- | --- | --- | --- | --- |
|  | **ZFO-2** | | **ZFO-3** | | **ZFO-4** | |
|  | **EDS-TEM** | **EELS** | **EDS-TEM** | **EELS** | **EDS-TEM** | **EELS** |
| **Zn** | 11.13 | 11.3 | 7 | 7.1 | 4.11 | 4.8 |
| **Fe** | 42.20 | 46.3 | 26.27 | 29.4 | 26.07 | 35.3 |
| **O** | 46.67 | 42.4 | 66.72 | 64 | 69.81 | 60 |
